# Abundant antibiotic resistance genes in rhizobiome of the human edible *Moringa oleifera* medicinal plant

**DOI:** 10.3389/fmicb.2022.990169

**Published:** 2022-09-15

**Authors:** Ashwag Y. Shami, Aala A. Abulfaraj, Mohammed Y. Refai, Aminah A. Barqawi, Najat Binothman, Manal A. Tashkandi, Hanadi M. Baeissa, Lina Baz, Haneen W. Abuauf, Ruba A. Ashy, Rewaa S. Jalal

**Affiliations:** ^1^Department of Biology, College of Sciences, Princess Nourah bint Abdulrahman University, Riyadh 11617, Saudi Arabia; ^2^Biological Sciences Department, College of Science and Arts, King Abdulaziz University, Rabigh 21911, Saudi Arabia; ^3^Department of Biochemistry, College of Science, University of Jeddah, Jeddah, Saudi Arabia; ^4^Department of Chemistry, Al-Leith University College, Umm Al Qura University, Makkah, Saudi Arabia; ^5^Department of Chemistry, College of Sciences and Arts, King Abdulaziz University, Rabigh, Saudi Arabia; ^6^Department of Biochemistry, Faculty of Science—King Abdulaziz University, Jeddah, Saudi Arabia; ^7^Department of Biology, Faculty of Applied Science, Umm Al-Qura University, Makkah, Saudi Arabia; ^8^Department of Biology, College of Science, University of Jeddah, Jeddah, Saudi Arabia

**Keywords:** resistome, horizontal gene transfer, antibiotic target, rhizosphere, microbiome, efflux pump, two-component system

## Abstract

*Moringa oleifera* (or the miracle tree) is a wild plant species widely grown for its seed pods and leaves, and is used in traditional herbal medicine. The metagenomic whole genome shotgun sequencing (mWGS) approach was used to characterize antibiotic resistance genes (ARGs) of the rhizobiomes of this wild plant and surrounding bulk soil microbiomes and to figure out the chance and consequences for highly abundant ARGs, e.g., *mtrA*, *golS*, *soxR*, *oleC*, *novA*, *kdpE*, *vanRO*, *parY*, and *rbpA*, to horizontally transfer to human gut pathogens *via* mobile genetic elements (MGEs). The results indicated that abundance of these ARGs, except for *golS*, was higher in rhizosphere of *M. oleifera* than that in bulk soil microbiome with no signs of emerging new soil ARGs in either soil type. The most highly abundant metabolic processes of the most abundant ARGs were previously detected in members of phyla Actinobacteria, Proteobacteria, Acidobacteria, Chloroflexi, and Firmicutes. These processes refer to three resistance mechanisms namely antibiotic efflux pump, antibiotic target alteration and antibiotic target protection. Antibiotic efflux mechanism included resistance-nodulation-cell division (RND), ATP-binding cassette (ABC), and major facilitator superfamily (MFS) antibiotics pumps as well as the two-component regulatory kdpDE system. Antibiotic target alteration included glycopeptide resistance gene cluster (vanRO), aminocoumarin resistance parY, and aminocoumarin self-resistance parY. While, antibiotic target protection mechanism included RbpA bacterial RNA polymerase (rpoB)-binding protein. The study supports the claim of the possible horizontal transfer of these ARGs to human gut and emergence of new multidrug resistant clinical isolates. Thus, careful agricultural practices are required especially for plants used in circles of human nutrition industry or in traditional medicine.

## Introduction

*Moringa oleifera* (or the miracle tree) is a drought-tolerant wild plant species of the family Moringaceae (Olson and Flora of North America Editorial Committee, 2010). This species is native to several habitats in Southern America, Africa and Asia including Saudi Arabia (Al-Eisawi and Al-Ruzayza, 2015). This plant is commonly used in traditional herbal medicine due to its health benefits as it possesses anti-fungal, anti-viral, anti-depressant as well as anti-inflammatory properties (Olson and Flora of North America Editorial Committee, 2010). Seed oil and leaf powder of *M. oleifera* contain a variety of useful proteins, vitamins (A, B1, B2, B3, B6, and C) and minerals (e.g., Ca, K, Mg, Fe, P, and Zn) (Gopalakrishnan et al., 2016). The plant is also used in treating edema, asthma and diabetes, in reducing high blood pressure, in treating stomach complaints, in fighting against bacterial diseases and cancer, in making bones healthier, and in healing skin wounds (Gopalakrishnan et al., 2016).

Recent advances in next generation sequencing (NGS) technology and subsequent bioinformatics approaches allow the accurate detection of microbial composition and function in the phyllospheric and rhizospheric regions of the plant (Vorholt, 2012; Bai et al., 2015; Vorholt et al., 2017). The rhizospheric region is regarded as a hotspot of effective microbial activity, recovery of new antibiotics and potential transfer of antibiotic resistance genes (ARGs) (Blau et al., 2019). NGS approaches of amplicon (or 16S rRNA) and metagenomic whole genome shotgun sequencing (mWGS) are used to decipher microbial composition and predicted function. Advantages of the latter approach include the ability to study the genomes of archaea, bacteria, fungi, and viruses and reaching accurate insights into taxonomy, biodiversity, phylogenetic relationships, and functionality of microbiomes (Segata et al., 2011). mWGS also allows studying the influences of environmental conditions and host-microbe interactions in shaping microbiome signatures and allows deciphering new ARGs and antibiotics in the phyllospheric and rhizospheric regions of a given plant especially if this plant is edible by human or livestock (Tringe et al., 2005; Raes et al., 2007).

Identification and development of new antibiotics was for years a top research priority among pharmaceutical companies (Dutescu and Hillier, 2021). However, the treatment of pathogenic bacteria is increasingly hindered by the ability of bacteria to generate resistance against antibiotics due to occurrence of gene mutations, or due to a bacterial taxon being transformed with ARGs of other bacterial taxon; a process called horizontal gene transfer (HGT) (Bäckhed et al., 2015). The latter incidence raises a concern that ARGs integrated in mobile genetic elements (MGEs) of rhizospheric or phyllospheric bacteria can contaminate an edible plant, thus, can eventually be transmitted to human gut microbiome. If a certain microbial pathogen exists in the latter environment, then a risk of acquired resistance against clinical antibiotics is raised. Up to date, no enough attention was paid to the serious incidence of horizontal transfer of ARGs existing in plant microbiomes to human gut or skin microbiome (Chen et al., 2019). In addition, the influence of resistome of native or wild plants was almost neglected (Obermeier et al., 2021). The study of rhizobiome of native plants allows the detection of natural antibiotics along with new versatile ARGs due to the existence of highly diversified microbial community in this dynamic region.

In the present study, we have deciphered abundant ARGs and predicted their metabolic processes that exist in rhizobiome of the native wild plant *Moringa oleifera* in order to detect the risks on human health due to the possible occurrence of HGT if organs of this plant were used by human either in human nutrition or herbal medicine.

## Materials and methods

### Sample collection and deoxyribonucleic acid extraction

Microbial samples were collected in three replicates from rhizosphere of *M. oleifera* plant grown naturally in the North Western region of Mecca district of Saudi Arabia near the red sea coast (21°12′17.8″N 39°31′26.4″E) and from surrounding bulk soil (Al-Eisawi and Al-Ruzayza, 2015). The selected three plants for the experiment are single-grown, have similar size and received no rainfall for > 3 months prior sample collection, while bulk soil samples were concurrently taken ≥ 10 m apart from the plants. For soil rhizospheric samples, plant lateral roots were first cut at ∼10–30 cm depth. Then, soil of a maximum of 1 cm apart from the root, not the soil that physically adheres to the root, was collected and immediately put in liquid nitrogen, transported to the lab, and stored at −20°C until use (Hurt et al., 2001).

### Whole genome shotgun sequencing and bioinformatics analysis

Deoxyribonucleic acid (DNAs) of different soil samples were extracted using CTAB/SDS method, and purity and integrity were checked by 1% agarose gel electrophoresis. DNA concentration was adjusted to 10 ng/μl using dsDNA Assay kit (Life Technologies, United States). Then, 1 μg of each DNA sample was shipped to Novogene Co. (Singapore) for whole metagenome shotgun sequencing. Physical fractionation of DNAs followed by data pre-processing were done and low quality bases (*Q*-value ≤ 38) exceeding 40-bp threshold were trimmed, and reads with N nucleotides over 10-bp threshold were removed. Then, effective clean data were sequenced on Illumina HiSeq 2500 platform after library preparation using an Ultra DNA Library Prep kit for Illumina (NEB, United States). Generated data were assembled to recover scaffolds using MEGAHIT (K-mer = 55) and chimeras were removed as described (Karlsson et al., 2012; Mende et al., 2012; Oh et al., 2014). Unassembled reads of different samples were mixed together to generate NOVO_MIX scaffolds exploring the less-abundant genes of the different samples. Generated scaffolds were cut off at “N” to get N-free fragments (or scaftigs) (Mende et al., 2012; Nielsen et al., 2014). Then, clean data were mapped to assembled scaftigs using Soap 2.21, and effective scaftigs (found in > 2 samples) were further utilized. Gene prediction was carried out by MetaGeneMark (Nielsen et al., 2014) and predicted genes were dereplicated using Cluster Database at High Identity with Tolerance (CD-HIT) (Li and Godzik, 2006; Fu et al., 2012). Then, non-redundant gene catalogs (nrGC) were constructed after removing redundancy using a greedy pairwise comparison (Li et al., 2014) and annotation was done using the binning reference-based classification method MEGAN (Huson et al., 2011, 2016).

All unique open reading frames (ORFs) were subjected to Blastp against CARD (Comprehensive Antibiotic Resistance Database)^1^ (*e*-value ≤ 1^*e*–5^) (Martínez et al., 2015) in order to identify and characterize ARGs. Then, relative abundance of different ARGs in unit ppm was estimated (Yang et al., 2013; Forsberg et al., 2014). ARGs were categorized to antimicrobial resistance (AMR) families (or metabolic processes) and resistance mechanisms were assigned as described (Liu and Pop, 2009). Based on abundance information of ARGs, heat map of abundance clustering for the top 30 highly abundant ARGs was drawn. Then, two circle charts were drawn to describe the overall proportion and distribution of the most redundant ARGs across the two soil types, on one hand, and the most redundant metabolic processes at the bacterial phylum level in ARO (antibiotic-resistant organisms) datasets, on the other hand. The first circle chart was divided into two parts of which the first refers to soil types (right side) and the second part refers to the most highly abundant ARGs (left side). The second chart was drawn to incorporate the most highly abundant bacterial phyla in terms of ARGs (right side) and the metabolic processes related to these highly abundant ARGs (left side). Stretches of different colors in the right side of inner circle refer to sum of relative abundance of all ARGs per sample in the first circus and sum of relative abundance of all metabolic processes per bacterial phylum in the second circus. While, color stretches in the left side refer to sum of relative abundance of all samples per ARG in the first circus and all bacterial phyla per metabolic process in the second circus. Colors in the right side of outer circle refer to detailed relative abundance of different ARGs per sample in the first circus and detailed relative abundance of different metabolic processes or AMR gene families per bacterial phylum in the second circus. While, colors in the left side refer to detailed relative abundance of different samples per ARG in the first circus and detailed relative abundance different bacterial phyla per metabolic process in the second circus.

### Validation of selected highly abundant antibiotic resistance genes *via* qPCR

Total RNAs were isolated from samples gathered for each soil type using RNA PowerSoil^®^ Total RNA isolation kit (Mo Bio, cat. no. 12866-25) following manufacturer instructions. DNA contamination was removed from RNA samples using RQ1 RNase-free DNase (Promega, Madison, WI, United States) and eventually checked by PCR. Then, primers of four highly abundant ARGs in plant rhizosphere in addition to those of 16S rRNA of *Bacillus subtilis*, used as a house-keeping gene, were designed using Netprimer software^2^ following standard criteria (Supplementary Table 1). Expression level of the different ARGs was detected by qPCR using Agilent Mx3000P System (Agilent technology, United States). Maxima™ SYBR Green/ROX qPCR was done as previously described (Bahieldin et al., 2015). Similar amounts of RNAs of the two samples were used and calculations were made to detect the expression level of ARGs relative to that of the housekeeping gene.

## Results

### Statistics of antibiotic resistance open reading frames

Non-redundant gene catalogs resulting from mWGS were subjected to Blastp with CARD (the Comprehensive Antibiotic Research Database) in order to detect and characterize ORFs referring to the ARGs and metabolic processes existing in the rhizobiome of *Moringa oleifera* and surrounding bulk soil microbiomes. Unique ORFs described in Supplementary Tables 2, 3 were generated either from assembled reads of a given soil sample or from scaftigs resulted from gathering and re-assembling unassembled reads of all samples (NOVO_MIX) referring to low abundant reads, respectively. The two tables described these ORFs, after being aligned with analog sequences in CARD, in terms of the mismatch and gap sizes (in nt) and nucleotides assigning gene start and end points. A total of 809 and 1,142 ORFs were generated from the two previously mentioned categories of gene assembly (Supplementary Tables 2, 3, respectively). Of which, a number of 1,416 ORFs across the two categories of gene assembly referring to a total of 183 ARGs were detected (Supplementary Table 4). The results in Supplementary Table 4 indicated that a number of 79 of these ARGs was represented by only a single ORF (referring to non-abundant ARGs), while the other ARGs were represented by two or more ORFs (referring to abundant, highly abundant, and the most highly abundant ARGs). Supplementary Figure 1 indicated the 30 most abundant ARGs referring to the top highly abundant ARGs with > 20 ORFs/ARG. Supplementary Table 4 indicates that the most highly abundant ARGs include *mtrA* (189 ORFs), *soxR* (104 ORFs), *oleC* (103 ORFs), *golS* (88 ORFs), and *novA* (74 ORFs) genes.

### Differential abundance of antibiotic resistance genes

**FIGURE 1:**
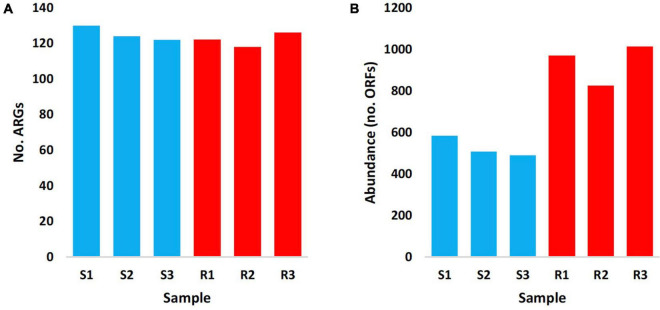
Number **(A)** and abundance (no. ORFs) **(B)** of antibiotic resistance genes (ARGs) of rhizobiomes (Rl–R3 in red) and bulk soil (Sl–S3 in blue) microbiomes surrounding *Moringa oleifera.*

**FIGURE 2:**
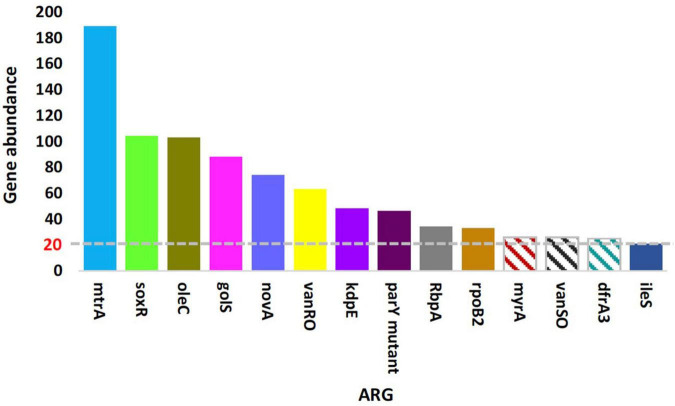
Means of the top highly abundant antibiotic resistance genes (>20 ORFs/ARG) across type of microbiome surrounding *Moringa oleifera.* Patterned columns refer to ARGs whose numbers of ORFs in bulk soil samples are non-homogeneous. Box colors of other ARGs match those of Figure 4.

The results in Figure 1 indicated almost no differences in the total number of ARGs in samples of the two soil type (average of ∼125 ARGs/sample), while abundance of these ARGs in terms of ORFs/ARG was extremely higher in rhizosphere of *M. oleifera* than that in bulk soil microbiome. Supplementary Table 4 indicates abundance of ARGs across types of microbiome surrounding *M. oleifera*, while Figure 2 refers to the most highly abundant ARGs with > 20 ORFs/ARG. Abundance of these 14 ARGs in samples within each soil type was homogeneous, except for three genes, e.g., *myrA*, *vanSO* and *dfrA3*, that showed non-homogeneous abundance of ORFs among bulk soil microbiome samples (Supplementary Table 5). These three ARGs were not analyzed further.

**FIGURE 3:**
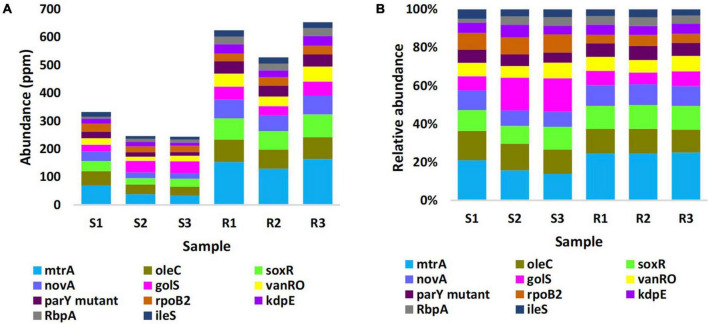
Abundance in unit ppm **(A)** and relative abundance **(B)** of the top highly abundant antibiotic resistance genes (>20 ORFs/ARG) in different samples of rhizobiomes (Rl–R3) and bulk soil (Sl–S3) microbiomes surrounding *Moringa oleifera.* Unit ppm was calculated by magnifying 10^6^ times of the original abundance data. Box colors of other ARGs match those of Figure 4.

**FIGURE 4:**
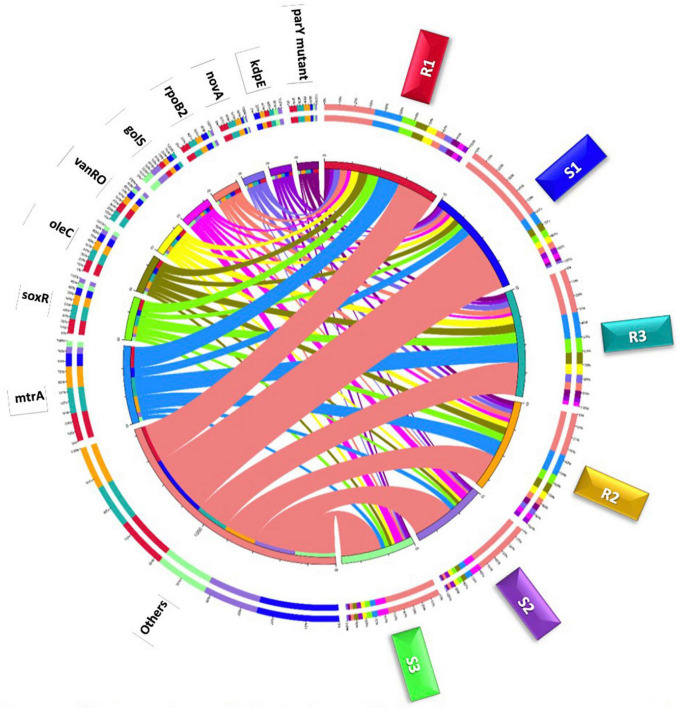
Circle chart of the top highly abundant antibiotic resistance genes (>20 ORFs/ARG) in different samples of rhizobiomes (Rl–R3) and bulk soil (S1–S3) microbiomes surrounding *Moringa oleifera.* The chart is divided into two sides, where the right one refers to samples (S and R) information and the left refers to antibiotic resistance genes (ARGs) information. The wideness of different scales of inner and outer circles refers to relative abundances of ARGs and samples. Colors in inner circle refer to sum of relative abundance of all ARGs per sample **(right side)** and sum of relative abundance of all samples per ARG **(left side)**. While, colors in outer circle refer to detailed relative abundance of different ARGs per sample **(right side)** and detailed relative abundance of different samples per ARG **(left side)**.

Estimated abundance (in unit ppm) of ARGs for the different samples of the two types of microbiomes is shown in Supplementary Table 5, while the most highly abundant ARGs (>20 ORFs/ARG) in microbiomes of the two soil types are shown in Figure 3A and described based on CARD information in Supplementary Table 6. The results in the table indicated that abundance of ARGs with > 20 ORFs/ARG was significantly higher in rhizobiome than that of bulk soil microbiome, except for *golS* gene, that showed no significant difference (*P* = 0.315) between samples of the two soil types. The results of relative abundance of ARGs supported those of abundance only for *mtrA*, *novA*, and *parY* mutant, as relative abundance of these three ARGs in rhizobiome is higher than that of bulk soil microbiome (Figure 3B). However, the results for *golS* (Pontel et al., 2007) and *rpoB2* (Ishikawa et al., 2006) genes showed higher relative abundance in bulk soil microbiome than that in rhizobiome (Figures 3B, 4). We assume the results of both abundance and relative abundance of ARGs ought to be considered.

### Differential abundance of bacterial phyla and antibiotic resistance mechanisms

**FIGURE 5:**
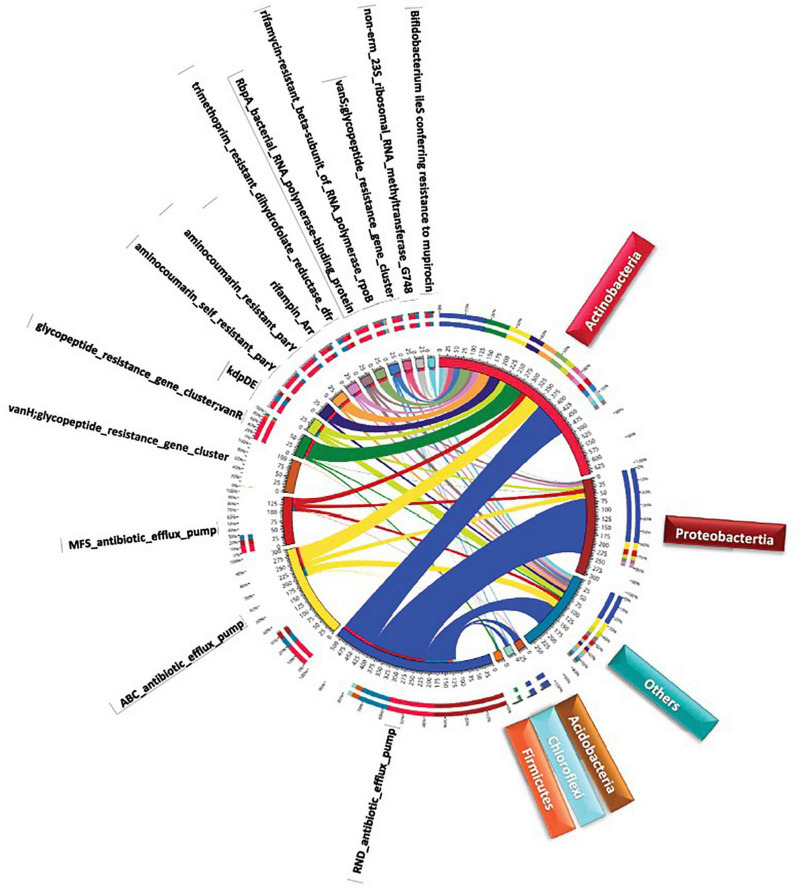
Circle chart of metabolic processes at the bacterial phylum level of the top highly abundant antibiotic resistance genes (>20 ORFs/ARG) in different samples of rhizosphere and bulk soil microbiome s surrounding *Moringa oleifera.* The chart is divided into two sides, where the right one refers to bacterial phylum information and the left refers to metabolic processes information. The wideness of different scales in inner and outer circles refers to relative abundances of metabolic processes and bacterial phylum. Colors in inner circle refer to sum of relative abundance of all metabolic processes per bacterial phylum **(right side)** ai1d sum of relative abundance of all bacterial phyla per metabolic process **(left side)**. While, colors in outer circle refer to detailed relative abundance of different metabolic processes per bacterial phylum **(right side)** and detailed relative abundance of different bacterial phyla per metabolic process **(left side)**.

ARGs were further categorized to metabolic processes or AMR families at the bacterial phylum level (Supplementary Table 7). The analysis was focused on 12 bacterial phyla and 55 categories of ARG-related metabolic processes. Based on abundance results, Figure 5 describes the five most common phyla, e.g., Actinobacteria, Proteobacteria, Acidobacteria, Chloroflexi, and Firmicutes, and the 13 most common metabolic processes. Abundance of phyla with > 30 ORFs regardless of metabolic process, abundance of metabolic processes regardless of phylum, as well as abundance of the interaction between phyla and metabolic processes are shown in Supplementary Figures 2, 3, and 5, respectively. Actinobacteria, Proteobacteria, Acidobacteria represent the most abundant bacterial phyla regardless of metabolic processes (Supplementary Figure 2). The eight most abundant metabolic processes shown in Supplementary Table 8 almost refer to all the previously detected highly abundant ARGs in Supplementary Table 6, except for *rpoB2* and *ileS* genes, whose metabolic processes, e.g., rifamycin-resistant beta-subunit of RNA polymerase (rpoB) and Bifidobacterium *ileS* conferring resistance to mupirocin, respectively, were less abundant (with < 30, Supplementary Figure 3), thus, were not analyzed further. These selected metabolic processes refer to three resistance mechanisms (Supplementary Table 8) namely antibiotic efflux, antibiotic target alteration and antibiotic target protection.

**FIGURE 6:**
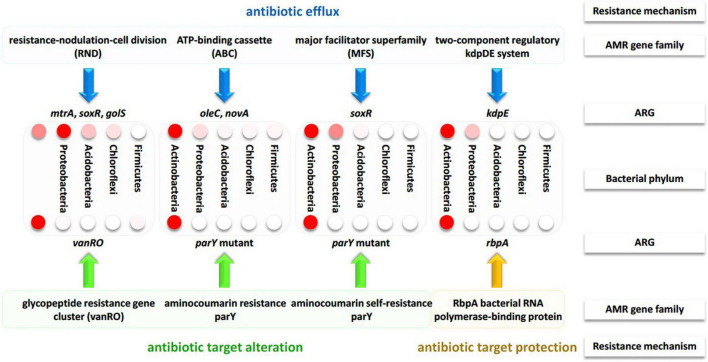
Summary of the resistance mechanisms along with the participating AMR gene families and their ARGs of bacterial phyla across soil microbiomes surrounding *Moringa oleifera.* Different intensities of the red color of the circles refer to the level of differential abundance in bacterial phyla.

AMR families referring to antibiotic efflux in this study include resistance-nodulation-cell division (RND), ATP-binding cassette (ABC), major facilitator superfamily (MFS) antibiotics as well as the two-component regulatory kdpDE (potassium dependent D/E) system. AMR families referring to antibiotic target alteration included glycopeptide resistance gene cluster (vanRO), aminocoumarin resistance parY and aminocoumarin self-resistance parY. While, AMR family referring to antibiotic target protection included RbpA bacterial rpoB-binding protein, only. As indicated in Supplementary Table 6, ARGs of *mtrA*, *soxR*, and *golS* participate in the metabolic process of RND antibiotic efflux, while *oleC* and *novA* genes participate in the metabolic process of ABC antibiotic efflux. *soxR* and *kdpE* genes participate in the metabolic processes of MFS antibiotic efflux and two-component regulatory kdpDE (potassium dependent D/E) system, respectively. Thus, *soxR* gene is the only participating in two metabolic processes. *vanRO*, *parY* mutant, and *rbpA* genes participate in the metabolic process of glycopeptide resistance gene cluster, in the metabolic process of aminocoumarin resistance/self-resistance, and in the metabolic process of RbpA bacterial rpoB-binding protein, respectively (Supplementary Table 6). The results of circle chart of Figure 5 referring to the left and right sides of inner circle support those of Supplementary Figures 2, 3 in terms of abundance, respectively. In terms of relative abundance, the results in Figure 5 (outer circle) and Supplementary Figure 4 indicate that Proteobacteria relatively dominates in terms of RND antibiotic efflux pump followed by Actinobacteria. However, the other seven metabolic processes are relatively dominated by Actinobacteria. Proteobacteria also showed high relative abundance of processes MFS antibiotic efflux pump and two-component regulatory kdpDE system. The results involving the most highly abundant ARGs, along with their metabolic processes and resistance mechanisms referring to the five bacterial phyla are summarized in Figure 6.

Validation of the resistomic data for the two soil types surrounding *M. oleifera* was done at the metatranscriptomic level *via* qPCR (Supplementary Figure 5). The experiment involved four highly abundant ARGs namely *mtrA* (acc. no. ARO:3000816), *soxR* (acc. no. ARO:3004107), *oleC* (acc. no. ARO:3003748), and *novA* (acc. no. ARO:3002522). Metabolic process of the first two ARGs is the RND antibiotic efflux pump, while that of the second two ARGs is the ABC antibiotic efflux pump. The results of qPCR for the metatranscriptomic data in Supplementary Figure 5 align with those of the *in silico* genomic dataset in Figure 3.

## Discussion

Native resistomes of the wild plants can provide unexplored antibiotic resistance mechanisms that add to our understanding of their contribution to the incidence of HGT (Berendonk et al., 2015). Obermeier et al. (2021) claimed that native plants likely harbor substantially diversified secondary metabolic processes that result in the recovery of a more diversified pristine resistome and antibiotics. Therefore, we thought it is important to explore native resistomes of naturally growing plants in the wild. Dissemination of ARGs is not only a concern within clinics, rather, this issue is prevalent in bacterial communities of the open environment (Chen et al., 2019). Initially, soil bacteria pose no direct threat to human health, while horizontal transfer of ARGs across different hosts to human pathogenic bacteria (*via* MGEs, e.g., plasmids, phages and integrons) can represent a major concern. Therefore, it is a demand to study resistome signatures and related resistance mechanisms in rhizobiomes of edible plants, and the factors that promote dissemination of ARGs.

Antibiotic resistance mechanisms generally include the antibiotic modifications, alteration of antibiotic target (decreasing the affinity of the binding site for the drug), antibiotic efflux, and global cell adaptations (Munita and Arias, 2016). Recent reports assign a new class of resistance mechanism known as antibiotic target protection, where a given resistance protein physically associates with an antibiotic target (or binding site) in the bacteria to hide it from the cognate antibiotic (Wilson et al., 2020). This resistance mechanism does not result in a permanent target modification, or requires a change of the nature of the target protein as the latter can be released again and becomes effective upon disappearance of the concerned antibiotic (Wilson et al., 2020).

The results in the present study indicated that interaction of *M. oleifera* roots with co-existing soil microbes did not result in the emergence of new soil ARGs (Figure 1), rather it affected differential abundance of existing ARGs as a consequence of differential abundance of microbes harboring these ARGs mostly in their MGEs. In accordance with the results of the present study, rate of resistance of pathogens to existing antibiotics was reported to be higher than that referring to the newly discovered ARGs (Boucher et al., 2009). Except for *golS* gene, abundance of ARGs in rhizobiome of *M. oleifera* was significantly higher than that of bulk soil microbiome (Figure 3). The main difference between microbiomes of bulk soil vs. those in the plant rhizosphere is the occurrence of plant exudation that participates in shaping structure and physiology of their respective rhizobiomes (Mendes et al., 2019). Exudation pattern mainly depends on plant genotype and developmental stage (Chaparro et al., 2014; Mönchgesang et al., 2016). Exudates can include minerals (e.g., carbon and nitrogen), simple molecules (e.g., sugars, organic acids, and secondary metabolites), and more complex polymers (e.g., mucilage) (Sasse et al., 2018).

In terms of relative abundance, *mtrA*, *novA*, and *parY mutant* genes showed higher level in rhizobiome ARGs than that in bulk soil microbiome, while those of *golS* and *rpoB2* genes showed opposite results (Figures 3, 4 and Supplementary Table 4). Across the highly abundant metabolic processes (with > 30 ORFs across phyla), Actinobacteria and Proteobacteria were the most abundant phyla followed by Acidobacteria, Chloroflexi, and Firmicutes (Figure 5, Supplementary Figure 2, and Supplementary Table 5). These metabolic processes refer to three resistance mechanisms namely antibiotic efflux, antibiotic target alteration and antibiotic target protection (Figure 6). Processes of antibiotic efflux pump included RND (Eswaran et al., 2004; Murakami et al., 2006; Blair and Piddock, 2009), ABC (Fath and Kolter, 1993), and MFS (Pao et al., 1998; Saier et al., 1999; Li and Nikaido, 2009), as well as the two-component regulatory KdpDE system (Freeman et al., 2013), while processes of antibiotic target alteration included glycopeptide resistance gene cluster for vancomycin resistance (vanRO) (Courvalin, 2006; Gudeta et al., 2014), and aminocoumarin resistance and self-resistant parY (Schmutz et al., 2003, 2004; Li and Heide, 2006), and those of antibiotic target protection included RbpA bacterial rpoB-binding protein, only (Newell et al., 2006; Hu et al., 2012; Figure 6). It was possible in the present study to connect highly abundant ARGs in samples of the two soil types (Figure 4) with their metabolic processes along with the bacteria promoting these processes (Figure 5). These connections are summarized in Figure 6.

### Resistance mechanisms in resistome of *Moringa oleifera*

#### Antibiotic efflux mechanisms

Efflux pump is an active transport protein with important role in bacterial homeostasis *via* expulsion of toxic substances or antibiotics (Fernández and Hancock, 2012). The efflux system results in the reduced accumulation of antibiotics and possibly results in multidrug resistance. Induction of efflux pump of bacterial antibiotics is an instant response of bacterial pathogens to potentiate their survival and resist toxic compounds during infectivity period. Efflux of toxins by bacteria ought to be fast in order to promote proper bacterial proliferation way before antibiotic therapy or host defense mechanisms are initiated (Koprivnjak and Peschel, 2011).

There are five bacterial efflux pumps classified into five families or metabolic processes. They are the resistance-nodulation-division (RND) family, the ABC family, the MFS, the small multidrug resistance (SMR) family, and the multidrug and toxic compound extrusion (MATE) family (Piddock, 2006; Poole, 2007; Sun et al., 2014). RND only exists in Gram-negative bacteria, mostly of phylum Proteobacteria, while other families are distributed among both Gram-positive and Gram-negative bacteria (Sun et al., 2014). Export of toxins by the different efflux pumps is energy-dependent, where ABC efflux pump relies on ATP hydrolysis, while the other pumps use proton motive forces for efflux (Chitsaz and Brown, 2017). The three efflux processes of RND, ABC, and MFS followed by the two-component regulatory KdpDE system seem to be dominating in rhizospheric region of *M. oleifera* due to the high abundance of ARGS of *mtrA*, *soxR*, *oleC*, *novA*, and *kdpE* (Figures 5, 6 and Supplementary Figure 3). While, efflux process of RND seems also to be dominating in bulk soil surrounding *M. oleifera* due to the high abundance of *golS*.

**FIGURE 7:**
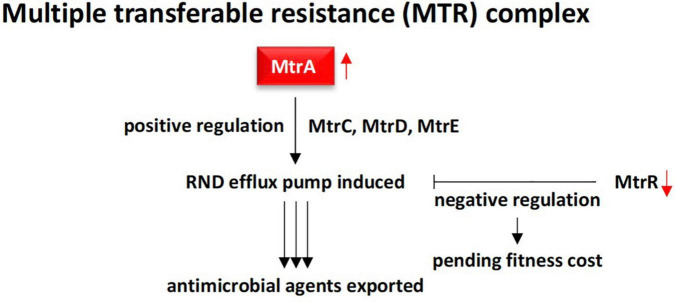
Demonstration of RND multidrug efflux system contributed by MtrA gonococcal protein (positive regulation) in rhizobiome of *M oleifera.* This efflux system is effective due to action of the downstream proteins MtrC, MtrD, and MtrE in addition to the absence of the MtrR transcriptional repressor. MtrR contributes to slower growth rate to maintain proper fitness cost.

RND superfamily transporters^3^ are made of large polypeptide chains located in the cytoplasm. RND efflux is composed of seven families including the hydrophobe/amphiphile efflux (or MtrCDE efflux) and the heavy metal efflux (HME) (Tseng et al., 1999). RND transporters are involved in maintaining cell homeostasis and in exporting a broad spectrum of toxic compounds and virulence determinants (Coyne et al., 2010). In the present study, the most abundant ARG in rhizobiome of *M. oleifera*, e.g., *mtrA* (multiple transferable resistance A) gene (Figures 3, 4), is known to encode a transcriptional activator or inducer of the MtrCDE multidrug efflux pump mostly in human pathogenic bacteria, e.g., *Neisseria gonorrhoeae* (Rouquette et al., 1999; Ohneck et al., 2011; Handing et al., 2018), and *Pseudomonas aeruginosa* (Du et al., 2018). MtrCDE efflux pump is a member of the highly abundant hydrophobic and amphiphilic efflux RND family. *N. gonorrhoeae* is the causative pathogen of gonorrhea and can also evade protective human immune responses (Wi et al., 2017; Nudel et al., 2018). MtrA is a gonococcal protein belonging to the AraC/XylS family that participates in the construction of the bacterial multiple transferable resistance (MTR) complex (Nikaido, 1996; Rouquette et al., 1999). The latter promotes an energy-dependent MtrCDE efflux pump comprising three cell envelope proteins namely MtrC, MtrD, and MtrE. These three proteins, in turn, serve in exporting antimicrobial agents outside the bacterial cell. These antimicrobial agents include hydrophobic agents (HAs), such as free fatty acids, bile salts, gonadol steroids, and antibacterial peptides (Handing et al., 2018). Many reports indicated that the MtrCDE efflux system can also act on antibiotics, like penicillin, erythromycin, cephalosporin, and rifampin (Olesky et al., 2006; Handing et al., 2018). On the other hand, HA-sensitive bacteria harbors another gene, e.g., *mtrR*, that otherwise encodes a transcriptional repressor of MtrCDE multidrug efflux pump system (Lucas et al., 1997). The latter gene does not exist in soil microbiome of *M. oleifera*, thus, does not seem to be involved in modulating the operon system of MtrCDE efflux pump in the resistome of *M. oleifera* (Figure 7). Ma et al. (1994) indicated that the loss of repressor activity is correlated with increased bacterial resistance to antimicrobial compounds. Thus, high abundance/relative abundance of *mtrA* gene (promoting positive regulation) and absence of *mtrR* gene (promoting negative regulation) in the resistome of *M. oleifera* can results in higher influence on virulence and the ability of the bacteria to resist antimicrobial agents more efficiently, in accordance with prior reports (Ohneck et al., 2011). This information indicates that horizontal transfer of such an *mtrA*^+^/*mtrR*^–^ resistance determinant to human gut can be dangerous. However, Handing et al. (2018) declared that bacterial acquisition of antibiotic resistance is associated with a fitness cost. Other reports claim that bacteria also need the negative regulation as over-activation of efflux pump can slow down rate of bacterial growth (Nikaido, 1996), thus, modulation of positive/negative regulation of the efflux pump is required for AMR, on one hand, and for optimal growth rate, on the other hand.

The *golS* gene encodes a mercury resistance (MerR)-like sensor, which is highly selective for gold (Au) ions (Brown et al., 2003; Pérez Audero et al., 2010). It controls proteins GolT, which is a P-type ATPase, and GolB, which is a small cytoplasmic metal-binding RND protein located in the inner membrane (Pontel et al., 2007). The latter two proteins participate in RND-dependent CBA efflux system that induces a resistance response against gold salts inside *Salmonella* cells. *Salmonella* is a poisonous bacterial disease that affects the intestinal tract and mostly infect human through contaminated water or food. The increase of GolS concentration could also result in direct sequestration of intracellular free Au levels (Brown et al., 2003). However, when high Au levels persists, CBA efflux system will be induced. The CBA efflux system is a tripartite protein complex consisting of subunits C, B, and A that generally direct efflux of metal ions or drugs from either the cytoplasm or the periplasm into the extracellular space. It was speculated that this efflux system likely results in transporting cellular metabolites (either damaged by Au or after forming a complex with this ion), rather than in transporting the toxic metal ion *per se* (Brown et al., 2003). As the efflux system involving *golS* gene is *Salmonella*-specific (Checa et al., 2007), it is expected to see in the present study that this gene is less abundant in the rhizospheric soil of *M. oleifera* (Figures 3, 4) where this microbe does not naturally interact with plant roots, thus *golS* gene is unlikely to be horizontally transferred through the plant system.

**FIGURE 8:**
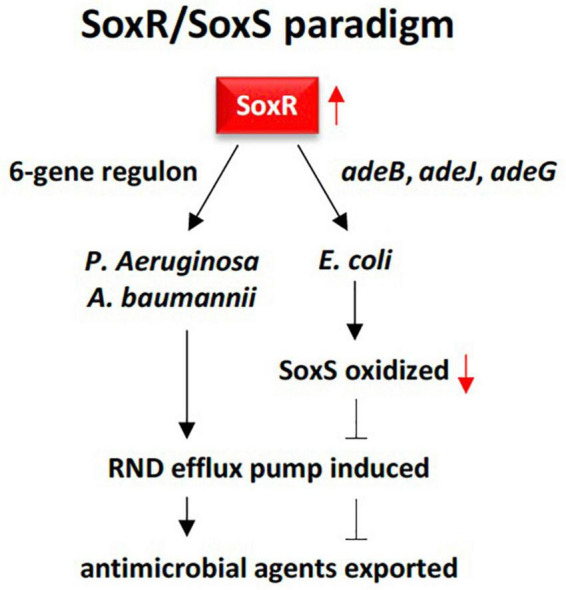
Demonstration of the RND multidrug efflux system contributed by SoxR response regulator in rhizobiome of *M oleifera* following two resistance patterns, one in *Escherichia coli*, and the other in *Pseudomonas aeruginosa and Acinetobacter baumannii.* The first pattern requires three genes namely *adeB, adeJ*, and *adeG* to oxidize SoxR that, in tum, oxidizes SoxS that eventually promotes the efflux system. The second pattern only requires a 6-gene regulon to be activated by SoxR in order to directly promote the efflux system. The first pattern unlikely apply to resistome in the rhizospheric region of *M. oleifera* as SoxS does not exist.

Recent reports indicated that *soxR* gene or transcription factor is a global RR that can directly or indirectly modulate bacterial multidrug resistance, growth and fitness (Palma et al., 2005; Li et al., 2017). SoxR promotes several efflux pump genes of the RND family including *adeB*, *adeJ*, and *adeG* (Marchand et al., 2004; Gu and Imlay, 2011). In alignment with the results of the present study, several reports indicated that *soxR* contributes to multidrug resistance mainly in members of the Gram-negative phylum Proteobacteria (Palma et al., 2005; Sakhtah et al., 2016). As an indirect regulator, SoxR protein is firstly oxidized in response to superoxide-generating agents in order to oxidize another protein namely *soxS* referring to the SoxR/SoxS paradigm (Pomposiello and Demple, 2001; Li et al., 2017). Although SoxR can be oxidized by extracellular superoxide anion, this anion might not be able to diffuse across the double membrane of the bacteria to oxidize SoxR. Instead, the latter can be rather oxidized by a permeable nitric oxide (NO) radical (Pomposiello and Demple, 2001). SoxS, in turn, promotes overexpression of a multidrug efflux machinery namely AcrAB-TolC in *Escherichia coli*, a member of the Proteobacterial family *Enterobacteriaceae*. AcrAB-TolC is a tripartite transporter that effluxes periplasm substrates out of the cell (White et al., 1997; Ruiz and Levy, 2014). This resistance pattern to resistome in the rhizospheric region of *M. oleifera* as SoxS does not exist. On the other hand, SoxR can directly regulate target genes, e.g., a 6-gene regulon, that promotes efflux pump in members of the Proteobacterial families *Pseudomonadaceae* (*Pseudomonas aeruginosa*) and *Moraxellaceae* (*Acinetobacter baumannii*) (Bialek-Davenet et al., 2011; Li et al., 2017). In particular, *A. baumannii* is a human pathogen that causes nosocomial and bloodstream infections (Peleg et al., 2008). SoxR was also reported to facilitate induction of MFS efflux pump genes in a way similar to that of the major facilitator MfsA (Saidijam et al., 2006; Dulyayangkul et al., 2016). The MFS system is composed of membrane transport proteins or facilitators that promote movement of solutes (e.g., drugs, metabolites, oligosaccharides, amino acids, etc.) across membranes as a consequence of chemiosmotic gradients (Marger and Saier, 1993). These facilitators open to either the extracellular or cytoplasmic side and simultaneously seal their opposing face to block bidirectional passage across the membrane, while only allow substrate entrance into the cell (Abramson et al., 2003). As *soxR* gene that promotes several efflux pump genes of the RND family is highly abundant in rhizobiome of *M. oleifera*, while *soxS* gene is absent (Figures 3, 4), then we expect that the *soxR*^+^/*soxS*^–^ resistance determinant can only represent a major risk on human health upon laterally transferred to gut microbiome in case *soxR* gene directly promoted efflux pump following the resistance pattern in either *P. aeruginosa* or *A. baumannii* (Figure 8).

The ABC transporter superfamily is composed of several membrane and membrane-associated (e.g., AAA ATPases) proteins that transport a variety of substrates (lipids, metabolic products, drugs, etc.) across extra- or intracellular membranes. Some of these proteins are involved in tumor resistance (Dean et al., 2001). During this ABC system, ATPases utilize ATP to provide energy required for the bidirectional translocation (influx/efflux) of substrates across membranes (Sauna et al., 2009). Of which, the OleC transporter of *Streptomyces antibioticus* is constituted by two proteins, e.g., OleC that recognizes, binds and hydrolyses ATP, and OleC5 that is a hydrophobic membrane protein (Olano et al., 1996). *oleC* gene, referred to as the *orf4* resistance determinant, shows similarity with proteins harboring ATP-binding domains, a feature of the ABC-transporter superfamily. This ARG dominates in the rhizospheric region of *M. oleifera* (Figure 3). The ABC transporter system allows secretion of oleandomycin out of the cell as an efflux transport mechanism of self-resistance against this antibiotic (Ma Rodriguez et al., 1993). Up to date, genus *Streptomyces* was reported to produce the majority of the clinical antibiotics (e.g., neomycin, cypemycin, grisemycin, bottromycins, chloramphenicol, aminocoumarin, etc.) mostly by *S. antibioticus* (de Lima Procópio et al., 2012) and *S. lividans* (Lee et al., 2007) that still can produce new antibiotics. Interestingly, *S. antibioticus* harbors another enzyme that catalyzes the last step of oleandomycin biosynthesis to release glucose from the inactivated oleandomycin and eventually generate new active antibiotics (Salas et al., 1994). In addition, *novA*, a highly abundant ARG in the present study (Figure 3), is an aminocoumarin resistance gene of such a biosynthetic gene cluster in *S. lividans* (Schmutz et al., 2003). The gene encodes a type III ABC-like transporter that confers a moderate level of resistance as this transporter presumably acts in the sequestration of the antibiotic into the medium (Méndez and Salas, 2001). The gene cluster harboring *novA* gene also contains two *gyrB* genes, e.g., *gyrB^S^* and *gyrB^R^* (Thiara and Cundliffe, 1989). Unlike *novA* gene, *gyrB^R^* presumably provides the principal resistance mechanism in the aminocoumarin antibiotic producer *Streptomyces lividans* due to the synthesis of coumarin-resistant topoisomerases (Schmutz et al., 2003). As abundance of *oleC* and *novA* genes of genus *Streptomyces* is high in the rhizosphere soil of *M. oleifera*, then, there is a chance that we can decipher new antibiotics as secondary metabolites generated by members of this genus that can be used further in clinical trials against newly emerging diseases. Fortunately, invasive infections with members of this genus are rarely encountered in clinical practice (Kapadia et al., 2007), thus, if their ARGs, like *oleC* and *novA* genes of rhizobiome of *M. oleifera*, were not horizontally transferred through contaminated food to other clinical species, it is unlikely that these bacteria can pose a risk to human health.

The two-component regulatory system (TCS) is ubiquitous and crucial in bacteria to maintain homeostasis and sense, thus, respond to changes in the surrounding environment (Cardona et al., 2018). TCS also responds directly in bacteria to the presence of antibiotics and acts as a major player in the realm of infectious diseases caused by pathogenic bacteria (Cardona et al., 2018; Tierney and Rather, 2019). Mechanisms of antibiotic resistance *via* TCS are divided into four categories of which the increased drug efflux dominates. The latter regulatory system comprise a histidine kinase (HK) for sensing environmental signals, and a response regulator (RR) for mediating cellular response and altering expression of target genes (Freeman et al., 2013). Examples include the membrane-bound HK and the cytosolic RR that form KdpD (HK)/KdpE (RR) TCS. This system refers to the regulation of the potassium (K^+^) dependent- (Kdp−) ATPase pump of operon KdpFABC. The gene encoding KdpE was proven to be highly abundant in the rhizobiome of *M. oleifera* (Figure 3). It was reported that KdpD/KdpE system can increase the ability of bacteria to cause the disease and/or survive in the host cell (Alegado et al., 2011). In *Salmonella typhimurium*, KdpD was proven to maintain bacterial growth in macrophage of nematode (*Caenorhabditis elegans*) cell lines as worms fed on the *kdpD* mutant strain lived longer than those fed on the wild type bacteria (Alegado et al., 2011). In terms of KdpE, prior results indicated its role as a RR of expression of a range of virulence genes in *Staphylococcus aureus* and *Enterohemorrhagic E. coli* (EHEC) (Hughes et al., 2009; Xue et al., 2011). This induced expression coincided with the increased survival rate of the two bacteria in human macrophages. Interestingly, deletion of kdpD/kdpE system in *S. aureus* resulted in the altered expression levels of > 100 genes regulating synthesis of surface protein and capsular polysaccharide, of alpha- and gamma-hemolysin toxins (offensive capabilities), and of metalloproteinase aureolysin that participates in inhibiting phagocytosis and killing the bacteria by neutrophils (defensive capabilities) (Laarman et al., 2011; Freeman et al., 2013). Moreover, *kdpE* gene was frequently reported to act as a RR gene in conferring resistance to the aminoglycoside antibiotic kanamycin *via* efflux pump system in *E. coli* (Hirakawa et al., 2003; Lv et al., 2021). Interestingly, KdpE is involved in two-compound systems with KdpD, QseC, or Cra, to confer pleotropic effects on the structure, and on the offensive and defensive capabilities of several bacteria including *Salmonella typhimurium*, *Staphylococcus aureus* and EHEC. Then, high abundance of *kdpE* gene in the rhizosphere soil of *M. oleifera* can pose very high risk to human health *via* contaminated food even if it is not transferred horizontally to pathogens of human gut.

#### Antibiotic target alteration and protection

**FIGURE 9:**
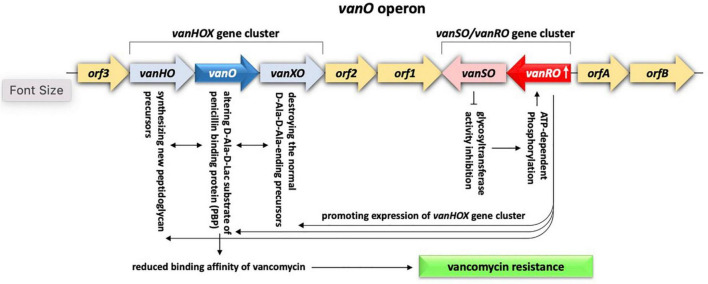
Demonstration of the antibiotic target alteration system existing in rhizobiome of *M. oleifera* and contributed by *vanRO* gene encoding the response regulator (RR) and *vanSO* gene encoding the histidine kinase (HK) of the VanRO/VanSO two-component system. The two genes represent a cluster of the *vanO* operon of which activated VanRO promotes expression of the other gene cluster of *vanO* operon namely *vanHOX* The latter cluster, in turn, acts in reducing binding affinity of vancomycin, thus, confers antibiotic resistance.

Resistance mechanism of alteration of antibiotic target refers to the ability of the bacteria to decrease the affinity for the drug by modifying its target site. The most common example is the glycopeptides (ex., vancomycin) that kill bacteria by inhibiting cell wall synthesis (Munita and Arias, 2016). Vancomycin resistance is common in *Enterococcus faecium*, a commensal microbe in the gastrointestinal tract of human that turned to be pathogenic causing serious diseases like neonatal meningitis or endocarditis (Arias and Murray, 2012). Vancomycin resistance occurs due to the action of one or more of the 11 *van* operons and acquisition of resistance in clinical isolates was previously proven to be due to horizontal transfer *via* MGEs, e.g., conjugative or non-conjugative plasmids carrying Tn*3* transposons (Arias and Murray, 2012). The clinical isolates of *E. faecium* harbor *vanA* and *vanB* operons that are either carried on plasmids or integrated in the bacterial chromosome (Munita and Arias, 2016). Vancomycin resistance is also common in *Rhodococcus equi*, a soil bacterium that can cause pneumonia in young foals and in immune-deficient adult humans (Franklin, 2016). The latter bacterium harbors *vanO* operon that includes gene cluster *vanSO*/*vanRO* acting as a TCS where VanSO is the HK and VanRO is the RR. *vanO* operon also includes a *vanHOX* gene cluster positioned opposite the *vanSO*/*vanRO* gene cluster in addition to five ORFs, e.g., ORFs1-3 and ORFsA-B, with no clear mode of action (Gudeta et al., 2014). The VanSO/VanRO system acts in promoting expression of the *vanHOX* gene cluster where initial sensing of the accumulation of substrates by VanSO inhibits glycosyltransferase activity, which, in turn, results in ATP-dependent phosphorylation of the RR VanRO. The latter, then, induces expression of the *vanHOX* gene cluster where *vanH* gene encodes a dehydrogenase enzyme necessary for the synthesis of new peptidoglycan precursors, *vanX* gene encodes an enzyme that destroys the normal D-Ala-D-Ala-ending precursors, while *vanO* gene encodes a ligase that synthesizes altered D-Ala-D-Lac substrate of penicillin binding protein (PBP) to reduce binding affinity of vancomycin and confer resistance (Figure 9) (Reynolds, 1989; Gudeta et al., 2014; Munita and Arias, 2016). *vanRO* gene is highly abundant in rhizobiome of *M. oleifera* (Figure 3). Being carried on a plasmid, the rhizospheric *vanRO* gene of *M. oleifera* along with its operon become a real threat of horizontal transfer to a human pathogen, thus, occurrence of acute diseases.

Topoisomerase IV (topo IV) is a type II topoisomerase involved in relaxing superhelical DNA prior replication and the decatenation of daughter chromosomes following DNA replication, e.g., two energetically driven processes by ATP hydrolysis (Schmutz et al., 2004). The topo IV enzyme is composed of the subunits ParC that contains the catalytic center for DNA cleavage/rejoining and ParE that contains the catalytic center for ATP hydrolysis. In Actinobacteria, e.g., *Streptomyces coelicolor* and *Bifidobacterium longum*, analogs of the *parC* and *parE* genes, e.g., *parX* and *parY*, exist. The latter type II topoisomerase genes exist in the biosynthetic gene cluster conferring aminocoumarin antibiotic resistance. This antibiotic is a potent inhibitor of the subunit B of the other drug target type II topoisomerase, namely gyrase (Maxwell, 1997). Members of genus *Streptomyces* harbor a modified *de novo* synthesized subunit of the drug target gyrase, namely aminocoumarin-resistant gyrase B to protect themselves from the inhibitory effects of aminocoumarin (Schmutz et al., 2004). The gene encoding this subunit, namely *gyrB^R^*, exists in the aminocoumarin gene clusters. In addition, there is an additional resistance gene, namely *parY^R^* or *parY* mutant, which is an analog of *parY* also exists. These modified versions of bacteria harboring GyrB*^R^* and ParY*^R^* are no longer targets for aminocoumarin antibiotics as they provide these bacteria with self-protection against the toxic effects of their own antibiotic, e.g., aminocoumarin. The mutant version of the *parY* gene is highly abundant in rhizobiome of *M. oleifera* (Figures 3, 4). Again, infection with members of the genus *Streptomyces* is rarely encountered in human clinical practice (Kapadia et al., 2007) and transfer of this ARG to clinical isolates of this genus almost represents no risk to human health, while horizontal transfer to pathogens of other bacterial phyla might pose a great risk.

**FIGURE 10:**
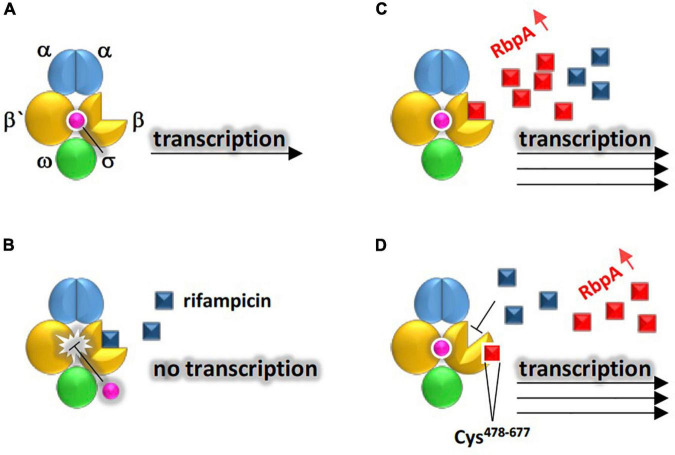
Demonstration of the two suggested resistance mechanisms existing in rhizobiome of *M. oleifera* against rifampicin conferred by RbpA to allow RNA polymerase (RNAP) properly conduct transcription of virulence genes. Structure of RNAP is made of the holoenzyme (representing the catalytic core that is made of five subunits of ααββ’ω) and a promoter-specific σ factor (for promoter recognition and conduction of transcription on the DNA antisense strand) located between residues Cys^478^ and Cys^677^ of β subunit. **(A)** No rifampicin, **(B)** presence of rifampicin and absence of RbpA, **(C)** resistance mechanism of antibiotic target protection where RbpA occupies rifampicin target site in β subunit of RNAP to make the binding site inaccessible to the antibiotic **(D)** resistance mechanism of alteration of antibiotic target where RbpA causes allosteric changes in the rifampicin binding site in RNAP.

In terms of antibiotic target protection system, bacteria prevent the antibiotic to reach its binding site (Newell et al., 2006; Wilson et al., 2020). Physical association of a resistance protein with the antibiotic target reduces the ability of the antibiotic to reach receptor of its target molecule. The *rbpA* (or RNA-polymerase binding protein A) gene is an ARG that is highly abundant in rhizosphere of *M. oleifera.* The encoded protein in *Mycobacterium tuberculosis* functions as a transcription activator that binds the core RNA-polymerase (RNAP), e.g., a target of the antibiotic rifampicin, to stabilize the principal promoter-specific housekeeping σ*^A^* factor, thus, to promote action of RNAP (Hu et al., 2012). Stimulation of σ*^A^* activity by RbpA is required for the expression of virulence genes and for pathogen proliferation in human macrophages (Hu et al., 2012). *M. tuberculosis* is the causative pathogen of tuberculosis (TB) in human of which rifampicin is the first-line drug used to cure the disease. It was suggested that RbpA binds cluster I of the β subunit of RNAP to prevent association/induce dissociation of the antibiotic and RNAP (Hu et al., 2012). The *rbpA* gene was first detected in *E. coli* (Maeda et al., 2000) and a year after in *Streptomyces coelicolor* (Paget et al., 2001). This gene was found to be highly upregulated during rifampicin treatment. Several resistance mechanisms were speculated for this gene of which we selected the two most appropriate for its action (Figure 10). They are the antibiotic target protection and antibiotic target modification. However, we assume that the first mechanism is likely to represent the *rbpA* gene action as the enzyme RNAP is still valid to cause transcription of the virulence genes after disappearance of the antibiotic. Figure 10 indicates that rate of expression by RNAP increases in the presence of RbpA compared with that in the absence of this protein (Hu et al., 2012). We expect that neither *E. coli* nor *Streptomyces coelicolor* can pose a serious health problem for human as RbpA is dispensable for survival of *S. coelicolor* (Newell et al., 2006). Rather, the problem rises if *rbpA* gene is horizontally transferred to clinical isolates of *M. tuberculosis* as the gene is, otherwise, essential for its survival (Forti et al., 2011). In addition, the *rbpA* encoded protein helps *M. tuberculosis* reaches the maximum expression levels of its virulence genes.

Plant rhizospheric soil is considered as the reservoir of ARGs due to the highly diversified microorganisms. Antibiotic resistance refers to the increase in the minimum inhibitory concentration of a compound (e.g., antibiotic) for a microbe (pathogenic or non-pathogenic) that was previously known to be sensitive at this antibiotic concentration (World Health Organization [WHO], 2012). Antibiotic resistance was hypothesized to be mainly the results of pre-existing environmental resistome. This hypothesis was proved when antibiotic-sensitive pathogens acquired resistance after introduction of new antibiotics and the gene *tetA*(P), for tetracyclin resistance, was proven to be a suitable biomarker of soil contamination with ARGs (Blau et al., 2018, 2019). When antibiotic substances exist in the rhizospheric region of a plant root, there might be evolutionary forces that pose a selection pressure on the plant rhizobiome and result in the emergence of microbes harboring ARGs. MGEs, such as viruses (e.g., viromes) or plasmids (e.g., plasmidomes), have uncovered the reason for rapidly disseminating antibiotic resistance within lineage of distant or even closely related microorganisms *via* HGT (World Health Organization [WHO], 2012). ARGs were questioned whether they are a result of gene shuffling/mutation or a result of resistance acquired through HGT (Nesme and Simonet, 2015). The latter is a genetic transformation process that was proven to promote the emergence of antibiotic resistant pathogenic bacteria (Khan et al., 2018). Recent reports interestingly proved that plant can also acquire exogenous microbial ARGs with the help of MGEs (Chen et al., 2017). Thus, MGEs can promote plant and microbial transformation processes with exogenous soil ARGs.

Plant-contaminating microbes that harbor ARGs and their vectors, e.g., MGEs are unlikely to be removed during food production, storage or processing, thus, can horizontally pass into microbiome of human gut. If the gut microbiome harbors pathogenic bacteria, then, the latter can acquire these ARGs, thus pose a great risk to human health (Chen et al., 2017, 2019). Human skin microbiome can also be exposed to antibiotic resistomes of phyllosphere and receive the same fate as that of gut microbiome (Chen et al., 2019). In addition, dead food-borne bacterial cells might lyse and existing extracellular ARGs consequently be disseminated in the environment (Levy-Booth et al., 2007). Examples of these extracellular DNAs include the self-transmissible plasmids conferring tetracycline resistance that were proven to be captured by *Escherichia coli* referring to the unintended transfer of this bacteria to human gut microbiome (Blau et al., 2018). Thus, HGT can refer to more than one layer of gene transfer within phyllosphere or rhizosphere microbiome of a plant species, e.g., from microbe in the rhizobiome to other microbe, from microbe to plant, from plant-contaminating microbes with ARGs to human microbiome (e.g., gut or skin), and from non-pathogenic to pathogenic microbes in either human organ. All of these layers of horizontal transfer of ARGs are mediated by the MGEs (Nardelli et al., 2012; Djordjevic et al., 2013).

In addition, concerns of pathogen resistance to antibiotic treatments were proven to be mostly limited to bacterial group namely ESKAPE (*Enterococcus*, *Staphylococcus*, *Klebsiella*, *Acinetobacter*, *Pseudomonas*, and *Escherichia*) (Boucher et al., 2009). Except for *Klebsiella*, ESKAPE group contains most of the selected highly abundant ARGs in the present study indicating that the transfer of these genes to analogs in the human pathogenic strains is possible. Cheng et al. (2012) indicated that the increase of antibiotic resistance in clinical pathogens is the result of gene mutation or fusion rather than the exchanges between soil and clinical resistomes, where only few resistance genes were shared between human gut and soil bacteria. Forsberg et al. (2014) reached similar conclusions and referred this to the limited rate of HGT between these two niches.

## Conclusion

In conclusion, the present study provides information on the structure of rhizospheric resistome of the wild plant *M. oleifera* and the possible ways and consequences of disseminating the soil ARGs in the open environment in order to develop interventions to prevent transfer of these ARGs to serious human pathogens or clinical isolates.

## Data availability statement

The raw data were deposited at the European Nucleotide Archive (ENA) (https://www.ebi.ac.uk/ena/browser/). Condition of deposit indicates that these raw data will be publicly available as soon as the article is published online. This is the standard condition with the deposit of nucleic acid sequences. Accession nos. (ERR10100770-74 and ERR10100781), sample no. (ERS12550318-23), and study no. (ERP139990).

## Author contributions

AS, RJ, and AA: conceptualization. HA: methodology. MR: software. NB, HB, and MT: validation. LB: formal analysis. AB and HB: investigation. MT: resources. NB: data curation. AS and MR: writing—original draft preparation. RA: writing—review and editing. AA: visualization. RJ: project administration and supervision. AS: funding acquisition. All authors read and agreed to the published version of the manuscript.

## Footnotes

1
https://card.mcmaster.ca

2
http://www.premierbiosoft.com/netprimer/index.html

3
https://en.wikipedia.org/wiki/Efflux_(microbiology)
