# Supplementary material for: Abundant antibiotic resistance genes in rhizobiome of the human edible Moringa oleifera medicinal plant
Source: Front Microbiol. 2022 Sep 15;13:990169. doi: 10.3389/fmicb.2022.990169 (PMC9524394; doi:10.3389/fmicb.2022.990169)
Supplement: Supplementary file 1 [file Data_Sheet_2.docx]

Figure S1. Heat map of the top 30 ARGs in terms of relative abundance of rhizobiomes (R1-R3) and bulk soil (S1-S3) microbiomes surrounding *Moringa oleifera*. Arrows refer to the top highly abundant antibiotic resistance genes with >20 ORFs/ARG.


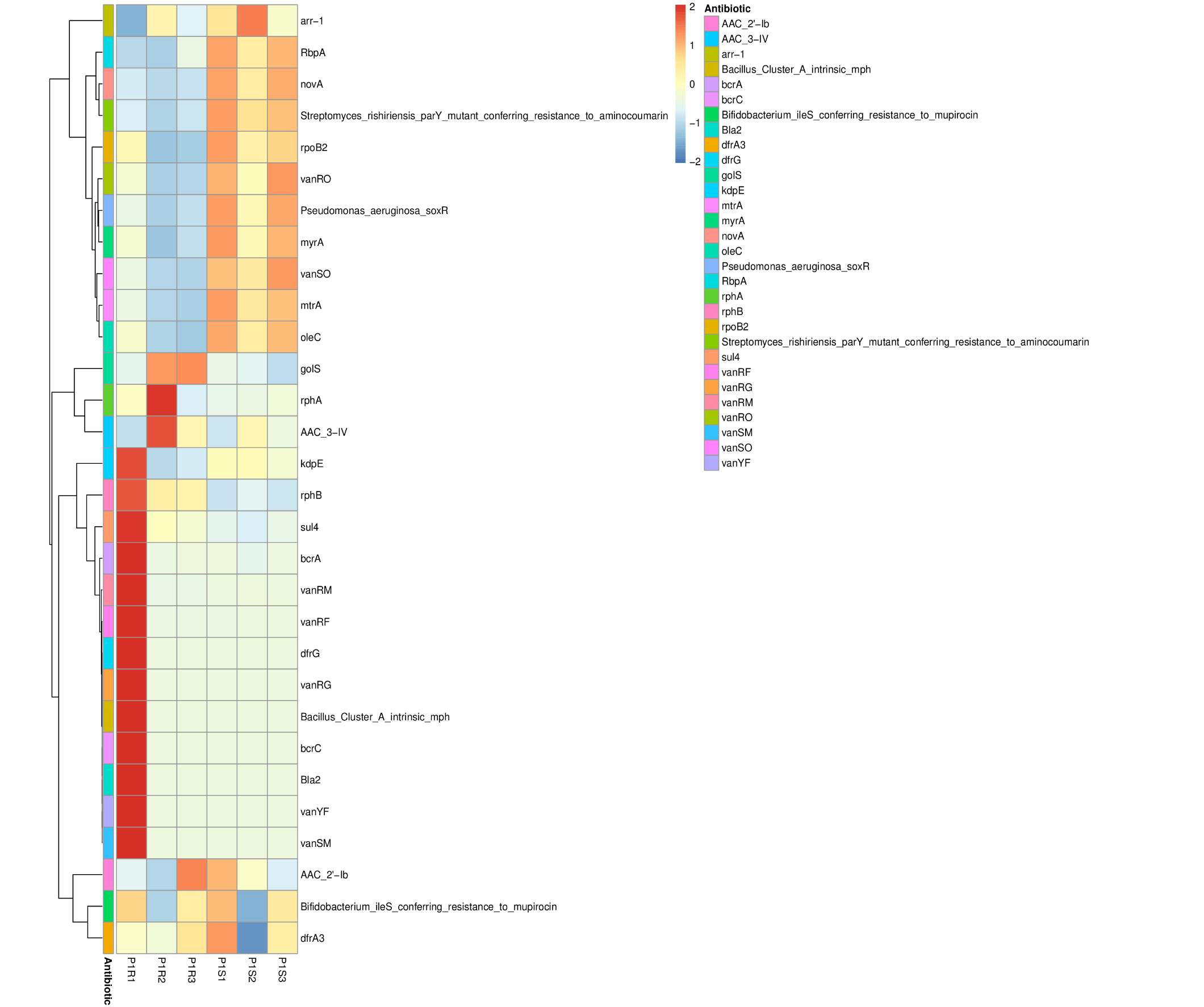


**R3**

**R2**

**R1**

**S3**

**S2**

**S1**

Figure S2. Abundance of bacterial phyla across metabolic processes involving the most highly abundant antibiotic resistance genes (>20 ORFs/ARG) across samples of rhizosphere and bulk soil microbiomes surrounding *Moringa oleifera*. Box colors of bacterial phyla match those in Figure 5.

Figure S3. Abundance of metabolic processes across bacterial phyla involving the most highly abundant antibiotic resistance genes across samples of rhizosphere and bulk soil microbiomes surrounding *Moringa oleifera*. Box colors of metabolic process match those of Figure 5. Horizontal line refers to the abundance threshold of processes (>30 ORFs) across phyla to be discussed further.

Figure S4. Relative abundance of metabolic processes within bacterial phyla involving the most highly abundant antibiotic resistance genes (>20 ORFs/ARG) in different samples of rhizosphere and bulk soil microbiomes surrounding *Moringa oleifera*. Box colors of bacterial phyla match those of Figure 5.

Figure S5. qPCR results of selected four highly abundant antibiotic resistance genes (ARGs), e.g., *mtrA* (accession no. ARO:3000816 in CARD), *soxR* (accession no. ARO:3004107 in CARD), *oleC* (accession no. ARO:3003748 in CARD) and *novA* (accession no. ARO:3002522 in CARD) for validating resistomic data of the two soil types surrounding *M. oleifera*. Metabolic process of the first two ARGs is the resistance-nodulation-cell division (RND) antibiotic efflux pump, while that of the second two ARGs is the ATP-binding cassette (ABC) antibiotic efflux pump. S = Bulk soil, R = rhizospheric soil. CARD = Comprehensive Antibiotic Resistance Database.
