# Supplementary material for: Abundant antibiotic resistance genes in rhizobiome of the human edible Moringa oleifera medicinal plant
Source: Front Microbiol. 2022 Sep 15;13:990169. doi: 10.3389/fmicb.2022.990169 (PMC9524394; doi:10.3389/fmicb.2022.990169)
Supplement: Supplementary file 2 [file Data_Sheet_1.ZIP › Supplementry data/Table S8.docx]

Table S8. Information retrieved from CARD site (<https://card.mcmaster.ca/ontology/>) for the top highly abundant ARG-related metabolic processes in samples of rhizobiomes and bulk soil microbiomes surrounding *Moringa oleifera*.

| **Process** | **Resistance-nodulation-cell division (RND) antibiotic efflux pump** |
| --- | --- |
| Accession | ARO:0010004 |
| Definition | Directed pumping of antibiotic out of a cell to confer resistance. Resistance-nodulation-division (RND) proteins are found in both prokaryotic and eukaryotic cells and have diverse substrate specificities and physiological roles. However, there are relatively few RND transporters and they are secondary transporters, energized not by ATP binding/hydrolysis but by proton movement down the transmembrane electrochemical gradient. |
| Resistance Mechanism | [antibiotic efflux](https://card.mcmaster.ca/ontology/36001) |
| Efflux Component | [efflux pump complex or subunit conferring antibiotic resistance](https://card.mcmaster.ca/ontology/36298) |
| Publications | Murakami et al. (2006); Eswaran et al. (2004); Blair and Piddock (2009) |
| **Process** | **ATP-binding cassette (ABC) antibiotic efflux pump** |
| Accession | ARO:0010001 |
| Definition | Directed pumping of antibiotic out of a cell to confer resistance. ATP-binding cassette (ABC) transporters are present in all cells of all organisms and use the energy of ATP binding/hydrolysis to transport substrates across cell membranes. |
| Resistance Mechanism | [antibiotic efflux](https://card.mcmaster.ca/ontology/36001) |
| Efflux Component | [efflux pump complex or subunit conferring antibiotic resistance](https://card.mcmaster.ca/ontology/36298) |
| Publications | Fath and Kolter (1993) |
| **Process** | **Major facilitator superfamily (MFS) antibiotic efflux pump** |
| Accession | ARO:0010002 |
| Definition | Directed pumping of antibiotic out of a cell to confer resistance. Major facilitator superfamily (MFS) transporters and ABC transporters comprise the two largest and most functionally diverse of the transporter superfamilies. However, MFS transporters are distinct from ABC transporters in both their primary sequence and structure and in the mechanism of energy coupling. As secondary transporters they are, like RND and SMR transporters, energized by the electrochemical proton gradient. |
| Resistance Mechanism | [antibiotic efflux](https://card.mcmaster.ca/ontology/36001) |
| Efflux Component | [efflux pump complex or subunit conferring antibiotic resistance](https://card.mcmaster.ca/ontology/36298) |
| Publications | Li and Nikaido (2009); Pao et al. (1998); Saier Jr et al. (1999) |
| **Process** | **Glycopeptide resistance gene cluster VanO** |
| Accession | ARO:3002918 |
| Definition | Homologous to vanA, contains a D-Ala-D-Lac ligase. The chromosome-located vanO gene cluster is inducible. Not much is known about the biochemistry about the vanO gene cluster. Gene orientation: orf1 RS orf2 HOX. |
| AMR Gene Family | [glycopeptide resistance gene cluster](https://card.mcmaster.ca/ontology/36373) |
| Drug Class | [glycopeptide antibiotic](https://card.mcmaster.ca/ontology/36220) |
| Resistance Mechanism | [antibiotic target alteration](https://card.mcmaster.ca/ontology/35997) |
| Publications | Gudeta et al. (2014) |
| **Process** | **kdpDE system** |
| Accession | ARO:3004046 |
| Definition | kdpDE is a two-component regulatory system in Escherichia coli, well studied for its role in potassium transport and homeostasis. kdpE is also implicated in virulence loci regulation and overexpression of kdpE is shown to confer resistance to aminoglycoside antibiotics. |
| Drug Class | [aminoglycoside antibiotic](https://card.mcmaster.ca/ontology/35935) |
| Resistance Mechanism | [antibiotic efflux](https://card.mcmaster.ca/ontology/36001) |
| Publications | Freeman et al. (2013) |
| **Process** | **Aminocoumarin resistant parY** |
| Accession | ARO:3000480 |
| Definition | Expression of parY(R), which encodes an aminocoumarin resistant topoisomerase IV, can confer aminocoumarin resistance. |
| Drug Class | [aminocoumarin antibiotic](https://card.mcmaster.ca/ontology/36242) |
| Resistance Mechanism | [antibiotic target alteration](https://card.mcmaster.ca/ontology/35997) |
| Publications | Schmutz et al. (2003); (2004) |
| **Process** | **Aminocoumarin self resistant parY** |
| Accession | ARO:3003787 |
| Definition | Inherent ParY resistant to aminocoumarin from an antibiotic producer. The presence of these genes confers self resistance to the antibiotic it produces. |
| Drug Class | [aminocoumarin antibiotic](https://card.mcmaster.ca/ontology/36242) |
| Resistance Mechanism | [antibiotic target alteration](https://card.mcmaster.ca/ontology/35997) |
| Publications | Schmutz et al. (2003); (2004) |
| **Process** | **RbpA bacterial RNA polymerase-binding protein** |
| Accession | ARO:3004243 |
| Definition | RbpA is a family of bacterial RNA polymerase-binding proteins, which acts as a transcription factor and binds to the sigma subunit of RNA polymerase. |
| Drug Class | [rifamycin antibiotic](https://card.mcmaster.ca/ontology/36296) |
| Resistance Mechanism | [antibiotic target protection](https://card.mcmaster.ca/ontology/35999) |
| Publications | Newell et al. (2006); Hu et al. (2012) |

**References**
