# Supplementary material for: Abundant antibiotic resistance genes in rhizobiome of the human edible Moringa oleifera medicinal plant
Source: Front Microbiol. 2022 Sep 15;13:990169. doi: 10.3389/fmicb.2022.990169 (PMC9524394; doi:10.3389/fmicb.2022.990169)
Supplement: Supplementary file 2 [file Data_Sheet_1.ZIP › Supplementry data/Table S6.docx]

Table S6. Information retrieved from CARD site (<https://card.mcmaster.ca/ontology/>) for the top highly abundant ARGs (>20 ORFs/ARG) in samples of rhizobiomes and bulk soil microbiomes surrounding *Moringa oleifera*.

| **CARD Short Name** | ***mtrA*** |
| --- | --- |
| Accession | ARO:3000816 |
| Definition | MtrA is a transcriptional activator of the MtrCDE multidrug efflux pump of Neisseria gonorrhoeae. |
| AMR Gene Family | [resistance-nodulation-cell division (RND) antibiotic efflux pump](https://card.mcmaster.ca/ontology/36005) |
| Drug Class | [penam](https://card.mcmaster.ca/ontology/36017), [macrolide antibiotic](https://card.mcmaster.ca/ontology/35919) |
| Resistance Mechanism | [antibiotic efflux](https://card.mcmaster.ca/ontology/36001) |
| Efflux Component | [efflux pump complex or subunit conferring antibiotic resistance](https://card.mcmaster.ca/ontology/36298) |
| Efflux Regulator | [protein(s) and two-component regulatory system modulating antibiotic efflux](https://card.mcmaster.ca/ontology/36590) |
| Publications | Rouquette et al. (1999) |
| **CARD Short Name** | ***oleC*** |
| Accession | ARO:3003748 |
| Definition | oleC is an ABC transporter isolated from Streptomyces antibioticus and is involved in oleandomycin secretion. |
| AMR Gene Family | [ATP-binding cassette (ABC) antibiotic efflux pump](https://card.mcmaster.ca/ontology/36002) |
| Drug Class | [macrolide antibiotic](https://card.mcmaster.ca/ontology/35919) |
| Resistance Mechanism | [antibiotic efflux](https://card.mcmaster.ca/ontology/36001) |
| Efflux Component | [efflux pump complex or subunit conferring antibiotic resistance](https://card.mcmaster.ca/ontology/36298) |
| Publications | Ma Rodriguez et al. (1999) |
| **CARD Short Name** | ***soxR*** |
| Accession | ARO:3004107 |
| Definition | SoxR is a redox-sensitive transcriptional activator that induces expression of a small regulon that includes the RND efflux pump-encoding operon mexGHI-opmD. SoxR was shown to be activated by pyocyanin. |
| AMR Gene Family | [major facilitator superfamily (MFS) antibiotic efflux pump](https://card.mcmaster.ca/ontology/36003), [resistance-nodulation-cell division (RND) antibiotic efflux pump](https://card.mcmaster.ca/ontology/36005) |
| Drug Class | [tetracycline antibiotic](https://card.mcmaster.ca/ontology/36189), [fluoroquinolone antibiotic](https://card.mcmaster.ca/ontology/35920), [penam](https://card.mcmaster.ca/ontology/36017), [glycylcycline](https://card.mcmaster.ca/ontology/35960), [rifamycin antibiotic](https://card.mcmaster.ca/ontology/36296), [cephalosporin](https://card.mcmaster.ca/ontology/35951), [phenicol antibiotic](https://card.mcmaster.ca/ontology/36526), [disinfecting agents and antiseptics](https://card.mcmaster.ca/ontology/43746) |
| Resistance Mechanism | [antibiotic efflux](https://card.mcmaster.ca/ontology/36001), [antibiotic target alteration](https://card.mcmaster.ca/ontology/35997) |
| Efflux Component | [efflux pump complex or subunit conferring antibiotic resistance](https://card.mcmaster.ca/ontology/36298) |
| Efflux Regulator | [protein(s) and two-component regulatory system modulating antibiotic efflux](https://card.mcmaster.ca/ontology/36590) |
| Publications | Palma et al. (2005); Sakhtah et al. (2016) |
| **CARD Short Name** | ***novA*** |
| Accession | ARO:3002522 |
| Definition | A type III ABC transporter, identified on the novobiocin biosynthetic gene cluster, involved in the transport and resistance of novobiocin. |
| AMR Gene Family | [ATP-binding cassette (ABC) antibiotic efflux pump](https://card.mcmaster.ca/ontology/36002) |
| Drug Class | [aminocoumarin antibiotic](https://card.mcmaster.ca/ontology/36242) |
| Resistance Mechanism | [antibiotic efflux](https://card.mcmaster.ca/ontology/36001) |
| Efflux Component | [efflux pump complex or subunit conferring antibiotic resistance](https://card.mcmaster.ca/ontology/36298) |
| Publications | Schmutz et al. (2003); (2004) |
| **CARD Short Name** | ***golS*** |
| Accession | ARO:3000504 |
| Definition | GolS is a regulator activated by the presence of golD, and promotes the expression of the MdsABC efflux pump |
| AMR Gene Family | [resistance-nodulation-cell division (RND) antibiotic efflux pump](https://card.mcmaster.ca/ontology/36005) |
| Drug Class | [phenicolantibiotic](https://card.mcmaster.ca/ontology/36526), [monobactam](https://card.mcmaster.ca/ontology/35923), [penam](https://card.mcmaster.ca/ontology/36017), [cephalosporin](https://card.mcmaster.ca/ontology/35951), [cephamycin](https://card.mcmaster.ca/ontology/35962), [carbapenem](https://card.mcmaster.ca/ontology/35939) |
| Resistance Mechanism | [antibiotic efflux](https://card.mcmaster.ca/ontology/36001) |
| Efflux Component | [efflux pump complex or subunit conferring antibiotic resistance](https://card.mcmaster.ca/ontology/36298) |
| Efflux Regulator | [protein(s) and two-component regulatory system modulating antibiotic efflux](https://card.mcmaster.ca/ontology/36590) |
| Publications | Pontel et al. (2007); Perez Audero et al. (2010) |
| **CARD Short Name** | ***vanRO*** |
| Accession | ARO:3002930 |
| Definition | Also known as vanRO, is a vanR variant found in the vanO gene cluster. |
| AMR Gene Family | [glycopeptide resistance gene cluster](https://card.mcmaster.ca/ontology/36373), [vanR](https://card.mcmaster.ca/ontology/36713) |
| Drug Class | [glycopeptide antibiotic](https://card.mcmaster.ca/ontology/36220) |
| Resistance Mechanism | [antibiotic target alteration](https://card.mcmaster.ca/ontology/35997) |
| Publications | Gudeta et al. (2014) |
| **CARD Short Name** | ***parY mutant*** |
| Accession | ARO:3000480 |
| Definition | Expression of parY(R), which encodes an aminocoumarin resistant topoisomerase IV, can confer aminocoumarin resistance. |
| AMR Gene Family | [aminocoumarin resistant parY](https://card.mcmaster.ca/ontology/36619) |
| Drug Class | [aminocoumarin antibiotic](https://card.mcmaster.ca/ontology/36242) |
| Resistance Mechanism | [antibiotic target alteration](https://card.mcmaster.ca/ontology/35997) |
| Publications | Schmutz et al. (2003); (2004) |
| **CARD Short Name** | ***rpoB2*** |
| Accession | ARO:3000501 |
| Definition | Due to gene duplication, the genomes of Nocardia species include both rifampin-sensitive beta-subunit of RNA polymerase (rpoB) and rifampin-resistant beta-subunit of RNA polymerase (rpoB2) genes, with ~88% similarity between the two gene products. Expression of the rpoB2 variant results in replacement of rifampin sensitivity with rifampin resistance. |
| AMR Gene Family | [rifamycin-resistant beta-subunit of RNA polymerase (rpoB)](https://card.mcmaster.ca/ontology/36349) |
| Drug Class | [rifamycin antibiotic](https://card.mcmaster.ca/ontology/36296) |
| Resistance Mechanism | [antibiotic target alteration](https://card.mcmaster.ca/ontology/35997) |
| Publications | Ishikawa et al. (2006) |
| **CARD Short Name** | ***kdpE*** |
| Accession | ARO:3003841 |
| Definition | kdpE is a transcriptional activator that is part of the two-component system KdpD/KdpE that is studied for its regulatory role in potassium transport and has been identified as an adaptive regulator involved in the virulence and intracellular survival of pathogenic bacteria. kdpE regulates a range of virulence loci through direct promoter binding. |
| AMR Gene Family | [kdpDE](https://card.mcmaster.ca/ontology/41098) |
| Drug Class | [aminoglycoside antibiotic](https://card.mcmaster.ca/ontology/35935) |
| Resistance Mechanism | [antibiotic efflux](https://card.mcmaster.ca/ontology/36001) |
| Efflux Regulator | [protein(s) and two-component regulatory system modulating antibiotic efflux](https://card.mcmaster.ca/ontology/36590) |
| Publications | Hirakawa et al. (2003); Freeman et al. (2013) |
| **CARD Short Name** | ***rbpA*** |
| Accession | ARO:3000245 |
| Definition | RNA-polymerase binding protein which confers resistance to rifampin. |
| AMR Gene Family | [RbpA bacterial RNA polymerase-binding protein](https://card.mcmaster.ca/ontology/41407) |
| Drug Class | [rifamycin antibiotic](https://card.mcmaster.ca/ontology/36296) |
| Resistance Mechanism | [antibiotic target protection](https://card.mcmaster.ca/ontology/35999) |
| Publications | Newell et al. (2006); Dey et al. (2011) |
| **CARD Short Name** | ***ileS*** |
| Accession | ARO:3000732 |
| Definition | Isoleucyl-tRNA transferase recognizes isoleucine codons and works with the ribosome to incorporate isoleucine amino acids into polypeptide chains. It can be targeted by antibiotics that have an analog of isoleucine through competitive inhibition. |
| Drug Class | [mupirocin](https://card.mcmaster.ca/ontology/36693) |
| Resistance Mechanism | [antibiotic target alteration](https://card.mcmaster.ca/ontology/35997) |
| Publications | Yanagisawa et al. (1994) |
